# Abdominal Manifestations of Lymphoma: Spectrum of Imaging Features

**DOI:** 10.5402/2013/483069

**Published:** 2013-09-02

**Authors:** Adonis Manzella, Paulo Borba-Filho, Giuseppe D'Ippolito, Marcella Farias

**Affiliations:** Hospital das Clínicas da Universidade Federal de Pernambuco, UFPE, Escola Paulista de Medicina, UNIFESP, Avenida Professor Moraes Rego, s/n, Cidade Universitária, 50670-420 Recife, PE, Brazil

## Abstract

Non-Hodgkin and Hodgkin lymphomas frequently involve many structures in the abdomen and pelvis. Extranodal disease is more common with Non-Hodgkin's lymphoma than with Hodgkin's lymphoma. Though it may be part of a systemic lymphoma, single onset of nodal lymphoma is not rare. Extranodal lymphoma has been described in virtually every organ and tissue. In decreasing order of frequency, the spleen, liver, gastrointestinal tract, pancreas, abdominal wall, genitourinary tract, adrenal, peritoneal cavity, and biliary tract are involved. The purpose of this review is to discuss and illustrate the spectrum of appearances of nodal and extranodal lymphomas, including AIDS-related lymphomas, in the abdominopelvic region using a multimodality approach, especially cross-sectional imaging techniques. The most common radiologic patterns of involvement are illustrated. Familiarity with the imaging manifestations that are diagnostically specific for lymphoma is important because imaging plays an important role in the noninvasive management of disease.

## 1. Introduction

 The malignant lymphomas, Hodgkin disease (HD) and non-Hodgkin lymphoma (NHL), comprise approximately 5% to 6% of all malignancies [1]. Lymphomas frequently involve nodal and extranodal structures in the abdomen and pelvis [2, 3].

HD is usually almost entirely confined to the lymph nodes [4, 5]. Extranodal lymphoma occurs in about 40% of patients and has been described in virtually every organ and tissue. In decreasing order of frequency, the spleen, liver, gastrointestinal tract, pancreas, abdominal wall, genitourinary tract, adrenal, peritoneal cavity, and biliary tract are involved [2].

The purpose of this review is to discuss and illustrate the spectrum of appearances of nodal and extranodal lymphomas, including AIDS-related lymphomas, in the abdominopelvic region using a multimodality approach, especially cross-sectional imaging techniques.

## 2. Nodal Disease 

**Figure 1:**
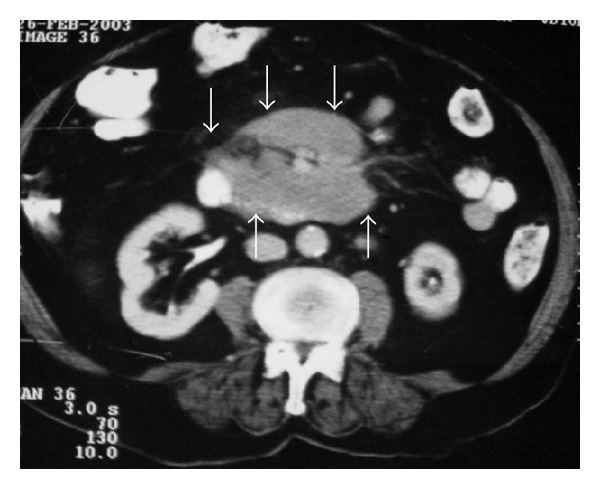
CT of the abdomen demonstrates lymph nodes involving mesenteric vessels (sandwich sign) anterior to aorta and inferior to vena cava in a patient with lymphoma (arrows).

Most normal lymph nodes (LN) are about 1 cm in size; however that size varies depending on their location [6]. Nodal disease can be solitary or more commonly multiple [3]. Solitary mass type of nodal lymphoma includes singular enlarged LN and fusion of multiple enlarged LN (Figure 1). CT usually shows a huge round mass or a lobular homogeneous density mass with uniform enhancement [3].

**Figure 2:**
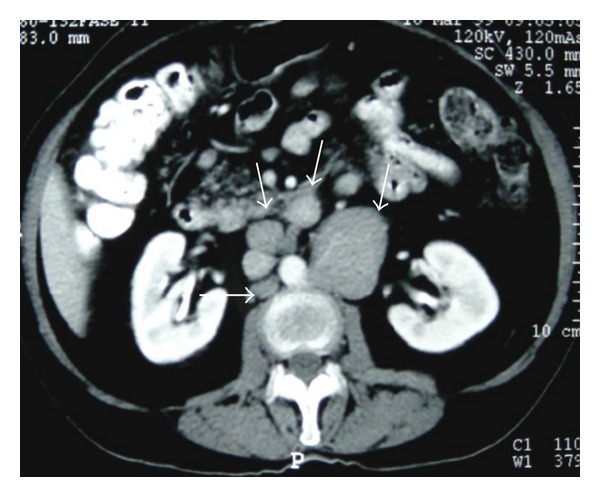
NHL. Axial contrast-enhanced abdominal CT shows retroperitoneal lymph nodes (arrows).

**Figure 3:**
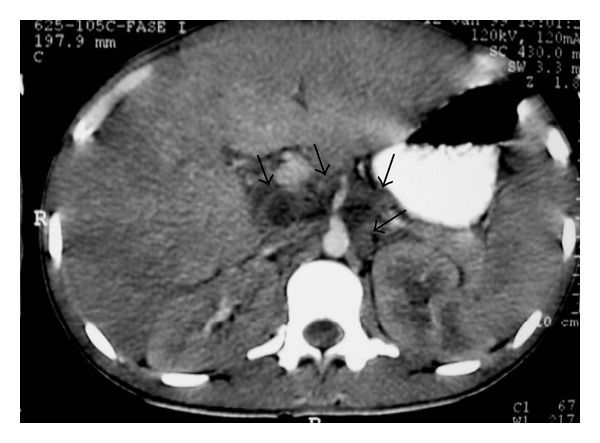
Nodal disease. Axial contrast-enhanced CT shows retroperitoneal lymph nodes (arrows).

**Figure 4:**
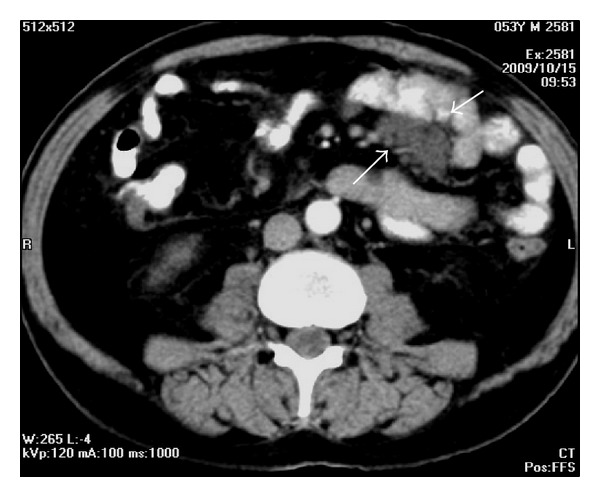
A 53-year-old man with lymphoma. Axial contrast-enhanced CT demonstrates mesenteric lymph nodes.

**Figure 5:**
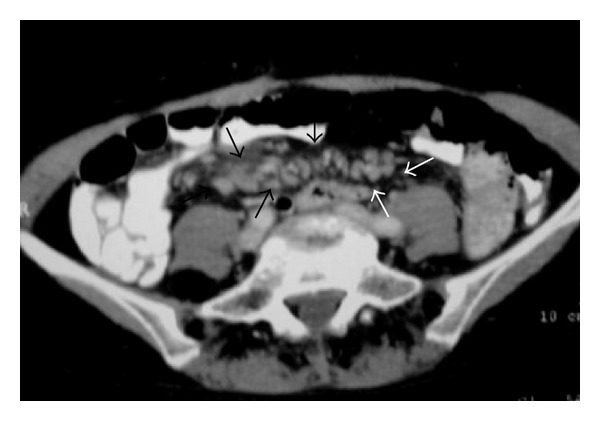
NHL in a 53-year-old woman. Axial pelvic contrast-enhanced CT shows mesenteric lymph nodes (arrows).

Multiple-nodular type of lymphoma, the most frequently seen, can be characterized by enlarged LN with regional distribution (Figures 2 and 3). Enlarged LN can be fused together and form a huge mass and can be seen on CT as uniform density lesions with mild homogenous enhancement [3]. Figures 4 and 5 illustrate mesenteric lymph nodes.

## 3. Hodgkin Disease versus Non-Hodgkin Lymphoma

**Figure 6:**
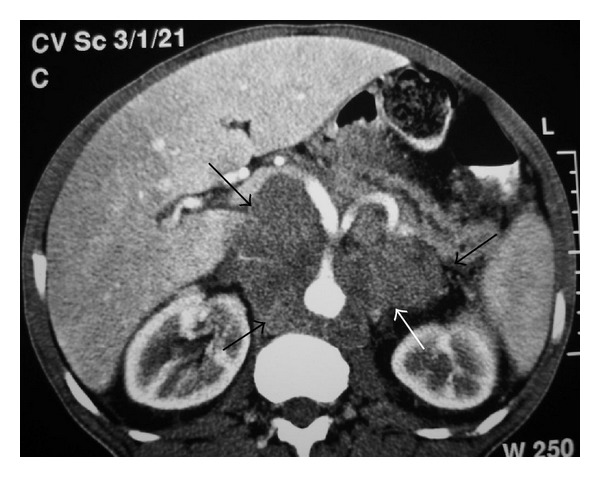
NHL. Axial contrast-enhanced abdominal CT shows retroperitoneal lymph nodes (arrows).

LN of HD are rarely seen in the mesentery (less than 5%–8.3%), with a small number of small lesions distributed dispersedly. NHL has a wide distribution including all sites of abdominal LN (Figure 6), and the mesentery is frequently involved (45%). The confluence of enlarged LN of HD is seldom seen (16.7%), which mainly occurs in multiple-nodular type (60%). However, the fusion of enlarged LN is common (60%) in NHL, showing vessel-imbedded signs, intestinal-imbedded signs, and cobblestone signs [3].

**Figure 7:**
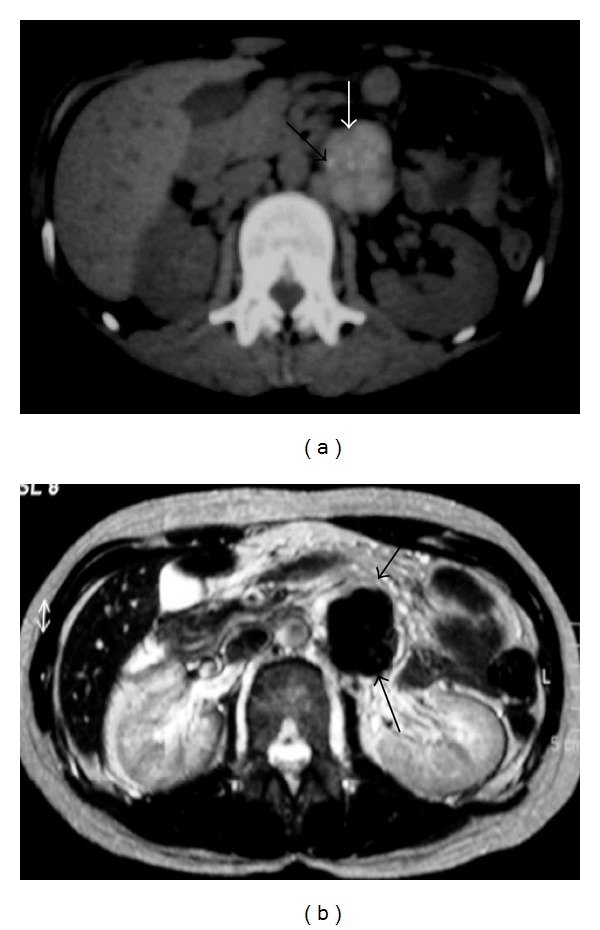
NHL. (a) Nonenhanced axial abdominal CT shows left paraaortic node (arrows) with small calcifications. (b) Axial T2-weighted image demonstrates left paraaortic hypointense mass (arrows).

The CT manifestations of nodal disease before and after radiochemotherapy are different, including changes in internal nodal characteristics. Increase in heterogeneous or rim enhancement of LN due to intranodular necrosis after treatment and calcifications of lesions may occur (Figure 7) [3].

## 4. Extranodal Lymphoma

Extranodal involvement is much less common in HD than in NHL. Extranodal invasion of adjacent tissue is seen in up to 15% of cases and hematogenous spread is found in 5%–10%. Extranodal involvement (except in the spleen and thymus) indicates stage IV HD. Contiguous disease, which requires local radiation therapy, must be distinguished from stage IV disease, which is treated with chemotherapy alone or combined with general radiation therapy. Also the extent of extranodal involvement must be evaluated because it is considered prognostic [5].

CT is the preferred modality, although ultrasonography and MR imaging may also be helpful. CT is preferred for evaluating hepatic lymphoma and diagnosing gastric lymphoma and renal or perirenal masses [5].

FDG positron emission tomography (PET) imaging has been shown to be an important technique for both staging and follow-up of nodal and extranodal lymphomas [4]. Recent studies indicate that PET/CT is superior to CT in detecting extranodal disease in the abdomen, especially in the spleen and liver [2, 4, 7, 8].

## 5. Spleen

The spleen is usually considered to be a “nodal organ” in HD and an extranodal organ in NHL [5]. It is involved in 20–40% of patients with lymphoma. Splenic involvement is found at initial presentation of lymphoma in 30–40% of patients with HD and in 10–40% of patients with NHL [7].

**Figure 8:**
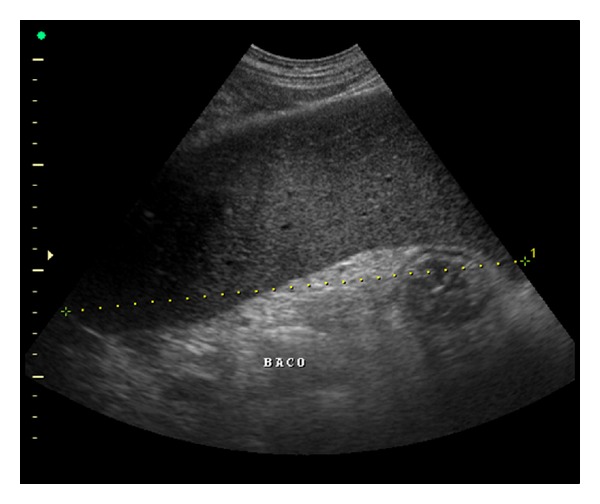
US of the spleen reveals splenomegaly.

The patterns of involvement include diffuse infiltration, with or without splenomegaly, and focal nodules [2]. Splenic involvement is typically diffuse, and only a small minority of cases manifest nodules larger than 1 cm in diameter. Diffuse infiltration may be present in spleens of normal size. Marked splenomegaly almost always indicates infiltration. However mild-to-moderate reactive splenomegaly (Figure 8) occurs in about 30% of patients in the absence of lymphoma deposits [5, 8]. 

### 5.1. Imaging

**Figure 9:**
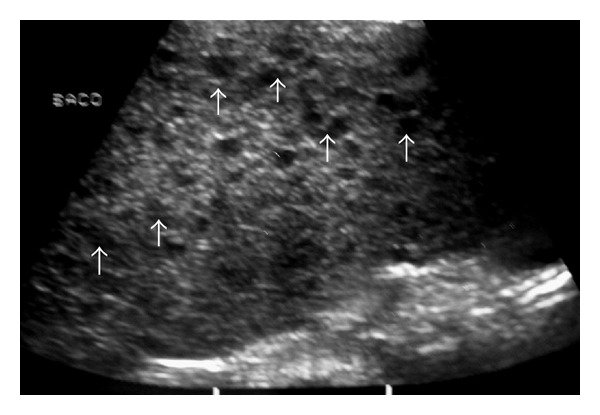
Young man with NHL. Sonogram of the spleen shows multiple hypoechoic nodules (arrows).

Nodules are characteristically hypoechoic at US (Figure 9), but very small deposits may not be detected.

**Figure 10:**
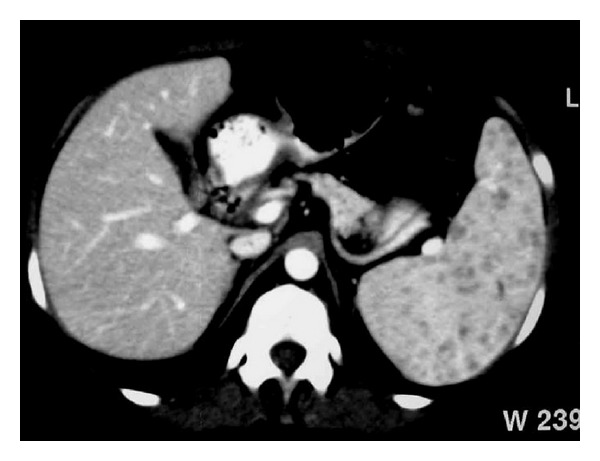
NHL. Axial contrast-enhanced CT image demonstrates multiple low-attenuation nodules in the spleen.

**Figure 11:**
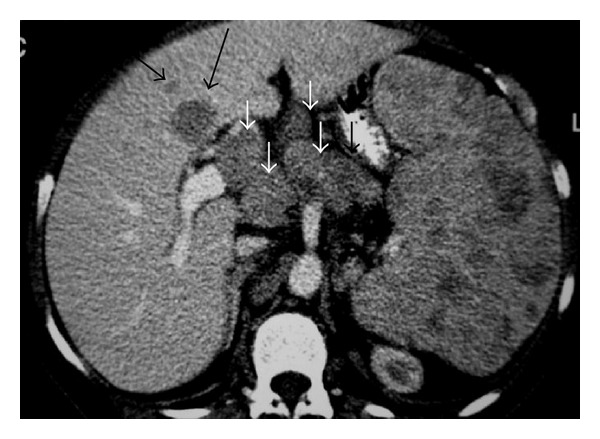
NHL. Axial contrast-enhanced CT image shows multiple low-attenuation splenic nodules. Note multiple enlarged LN (small arrows) and hepatic lesions (long arrows).

**Figure 12:**
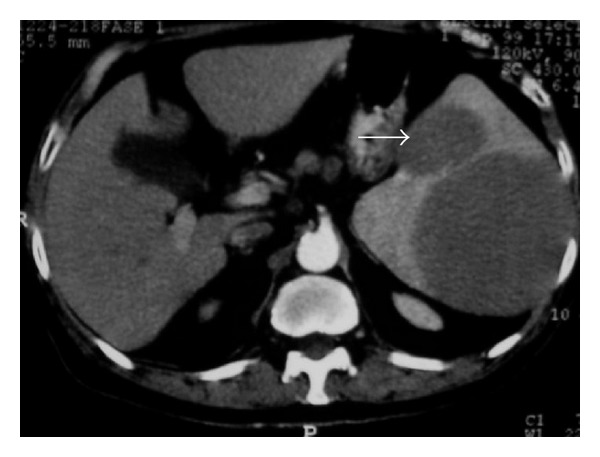
A 40-year-old woman with lymphoblastic lymphoma. Axial contrast-enhanced abdominal CT shows splenomegaly with multiple low-attenuation nodules (arrows).

**Figure 13:**
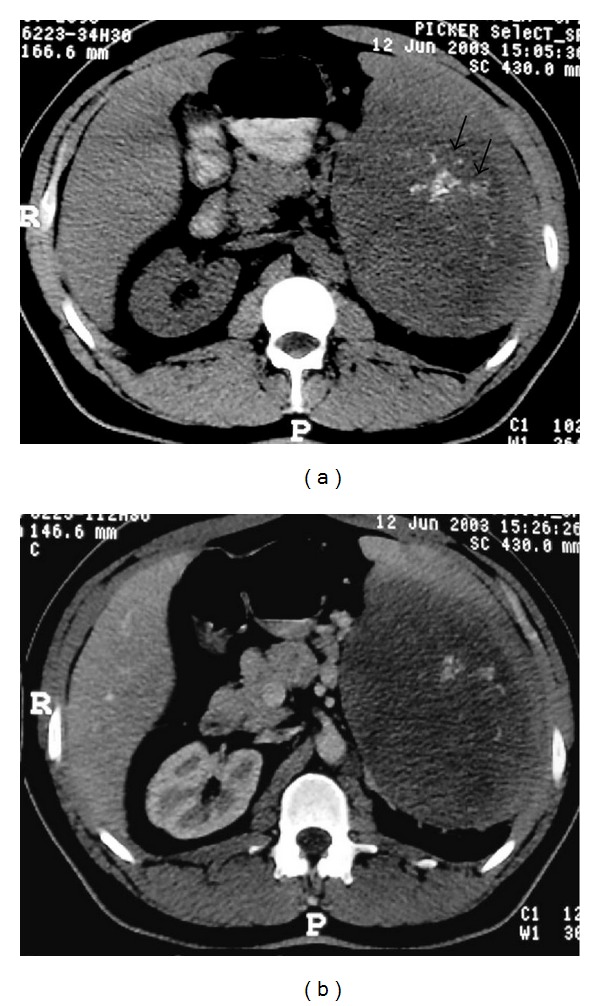
A 29-year-old man with NHL involving the spleen. Axial abdominal CT before (a) and after administration of intravenous CM (b) showing a large mass in the spleen (long arrows) with calcifications (small arrows).

**Figure 14:**
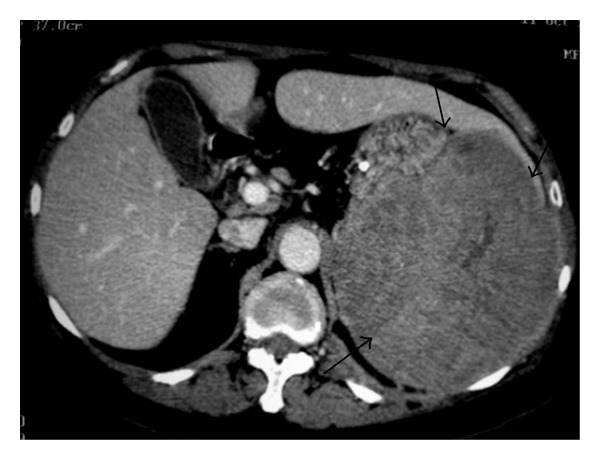
NHL involving the spleen. Axial abdominal CT after administration of intravenous CM reveals a large mass in spleen (arrows).

At CT, images demonstrate low attenuation (Figures 10, 11, 12, 13, and 14) with reduced contrast material (CM) enhancement compared with normal splenic tissue [5, 8]. 

At MR nodules are hypointense or isointense on T1-weighted images (T1-WI) and hyperintense on T2-WI and demonstrate reduced enhancement after administration of gadolinium (Gd) compared with normal spleen [2].

For initial staging of splenic involvement in malignant lymphoma, the sensitivity and specificity of PET/CT can reach 100% and 95%. The sensitivity of the combined approach is higher than that of either technique alone [7].

## 6. Liver

**Figure 15:**
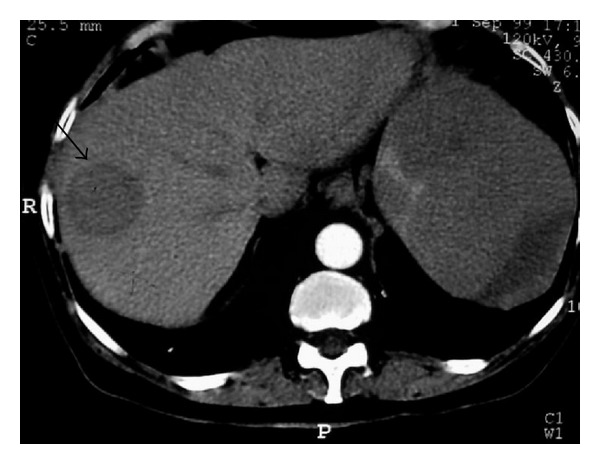
A 40-year-old woman with lymphoblastic lymphoma. Axial contrast-enhanced abdominal CT demonstrates circumscribed low-attenuation mass in the liver (arrow). Hypodense lesions are also seen in spleen.

**Figure 16:**
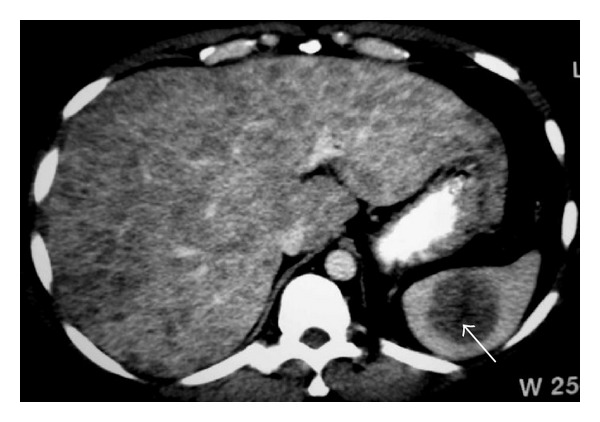
NHL. Axial contrast-enhanced CT image reveals multiple small low-attenuation nodules in the liver. Note also hypodense mass involving the spleen (arrow).

Primary hepatic HD is very rare [5]. However, secondary liver involvement is fairly common and is usually associated with lymph node disease. HD of the liver is almost invariably associated with disease of the spleen (Figure 15). Involvement of the liver occurs in up to 15% (6–20%) of patients with lymphoma and may be focal or diffuse with or without hepatomegaly [2, 5]. It is usually diffuse, with discrete nodular lesions being present in only 10% of cases. HD manifests more often as miliary lesions (<1 cm in diameter) than as masses (Figure 16) [5, 8]. 

### 6.1. Imaging

FDG PET/CT is more accurate than other cross-sectional techniques for the detection of diffuse hepatic involvement [2].

Focal hepatic lymphoma appears as circumscribed nodules that are hypoechoic and show no posterior acoustic enhancement on US.

**Figure 17:**
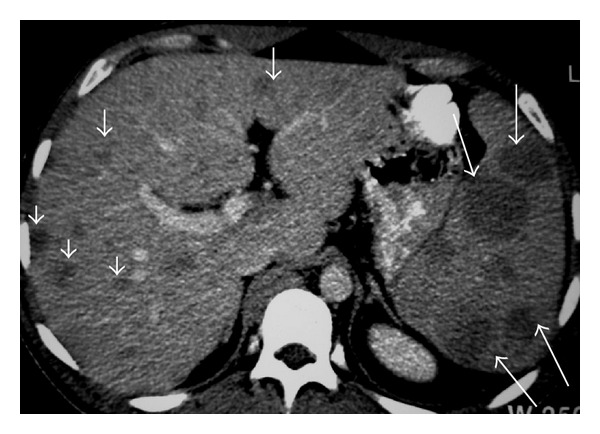
NHL. Axial contrast-enhanced CT image shows low-attenuation nodules in the liver (short arrows) and spleen (long arrows).

**Figure 18:**
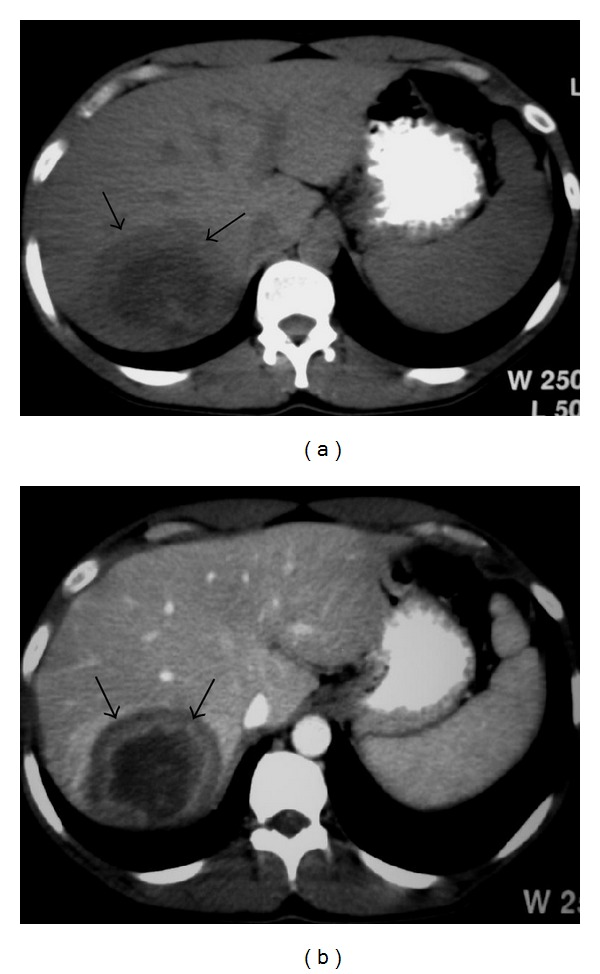
A 44-year-old man with NHL involving liver. (a) Nonenhanced axial CT of the liver reveals heterogeneous mass with central hypoattenuation (arrows). (b) Axial contrast-enhanced CT shows peripheral enhancement of the lesion (arrows).

**Figure 19:**
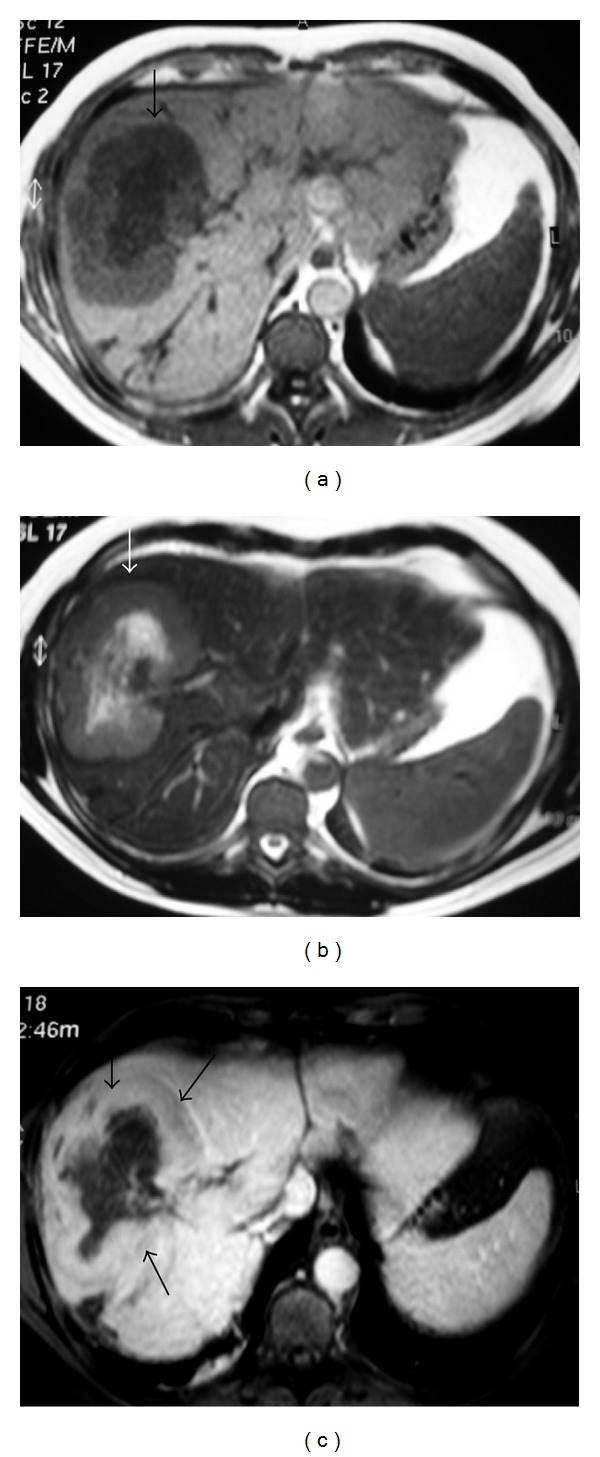
NHL. Axial liver MRI. (a) T1WI and (b) T2WI show heterogeneous mass (arrows) with central area of low signal on T1WI and high signal on T2WI (arrows). (c) The lesion shows enhancement.

On contrast-enhanced CT, the nodules are low attenuation (Figures 17 and 18), and on MRI, they may appear as hypointense or isointense compared with normal liver on T1-WI and as hyperintense on T2-WI (Figure 19) and may show reduced enhancement [2, 8].

## 7. Gastrointestinal Tract

HD rarely involves the gastrointestinal (GI) tract. Primary HD of the GI tract usually involves a single site. Multiple sites are rarely involved in disseminated HD [5].

Extranodal lymphoma in the GI tract occurs in 10–30% of all patients with NHL [9]. The stomach, small bowel, pharynx, large bowel, and esophagus are involved in decreasing order of frequency [2].

### 7.1. Stomach

**Figure 20:**
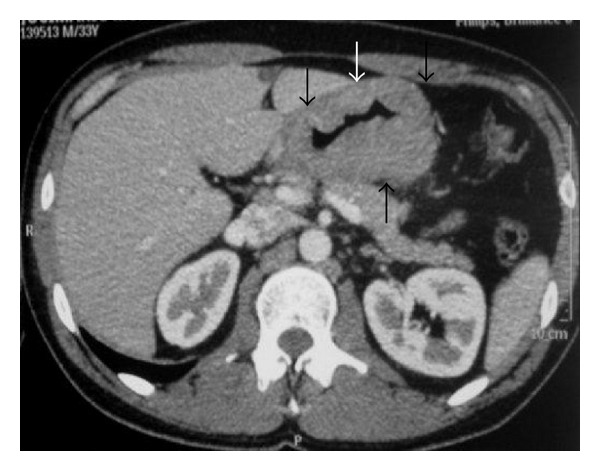
A 33-year-old man with gastric lymphoma. Axial contrast-enhanced abdominal CT demonstrates irregular thickening of the wall of the stomach (arrows).

The stomach is the most frequent site of malignant lymphoma of the GI tract (60–75% of cases) [9, 10]. However primary gastric HD is extremely rare. The patterns of gastric involvement include polypoidal mass, diffuse or focal infiltration, ulcerative lesion, or mucosal nodularity [2]. The infiltrating form is the most common (Figure 20) and may be difficult to differentiate from scirrhous carcinoma. CT demonstrates gastric wall thickening with a smoothly lobulated outer border [5].

### 7.2. Small Bowel Involvement

**Figure 21:**
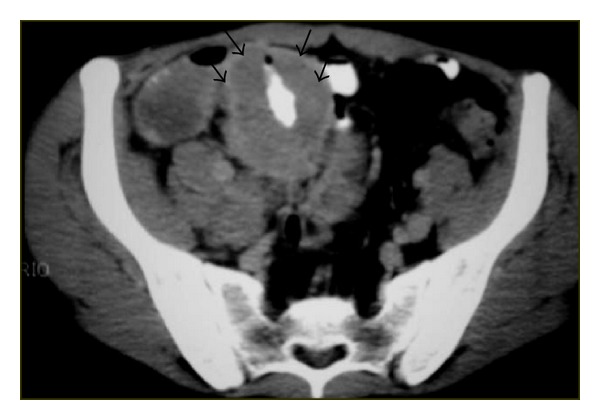
NHL. CT of the pelvis shows concentric thickening of small bowel loop on the right reducing its lumen (arrows).

Lymphoma is the most common malignancy of the small bowel, and in recent years its incidence related to B-cell hyperactivation in HIV-positive patients has increased. Primary NHL, Burkitt lymphoma, MALT-type lymphoma, and, rarely, HD have been described involving the small intestine [10]. The patterns of small bowel involvement include solitary or multiple nodules, circumferential wall thickening (Figure 21) with or without aneurysmal dilatation, and direct extension from mesenteric nodes [2, 10].

### 7.3. Large Bowel Involvement

**Figure 22:**
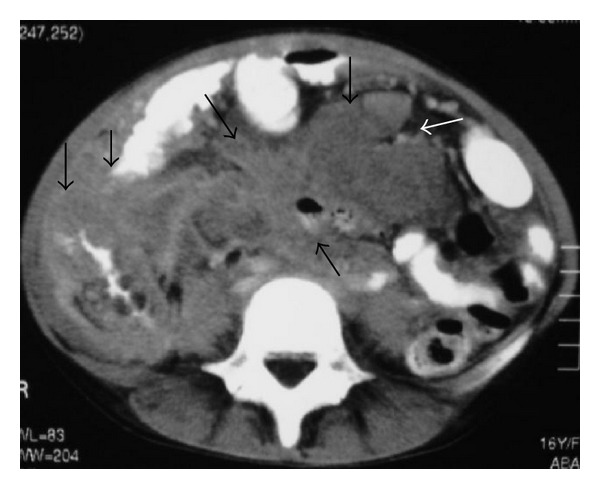
NHL. Axial CT shows masses involving small bowel loops and ascending colon (arrows).

**Figure 23:**
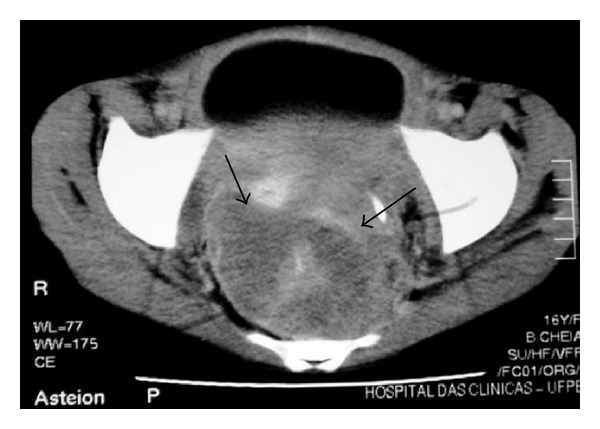
NHL in a 16-year-old girl. Axial contrast-enhanced CT of the pelvis reveals soft-tissue mass involving the rectal region (arrows).

**Figure 24:**
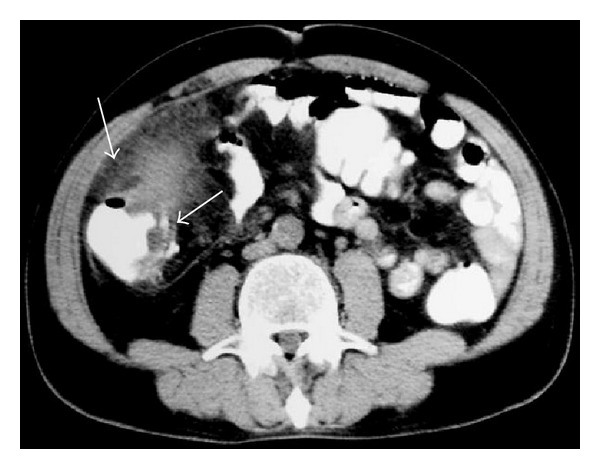
NHL. CT shows soft-tissue mass involving the ascending colon (arrows).

HD of the colon is uncommon [5, 10]. The cecum and rectum are most commonly involved [10]. The patterns of large bowel involvement include bulky polypoidal mass, infiltrative tumor, and aneurysmal dilatation (Figures 22, 23, and 24) [2]. In contrast to GI adenocarcinoma, lymphoma is more likely to involve multiple and longer segments of gut and is less likely to cause bowel obstruction.

MRI is used in local staging of rectal cancers. Lymphoma usually has homogeneous intermediate signal intensity on T1-WI, heterogeneous hyperintensity on T2-WI, and mild-to-moderate enhancement after Gd injection [10].

### 7.4. Appendix

Primary lymphoma of the appendix is also very rare, with only a few case reports in the literature, although it is more common to see cecal lymphoma extending to the base of the appendix [10, 11]. Appendiceal lymphoma may present clinically as acute appendicitis [11].

### 7.5. Pancreas

Pancreatic HD is extremely rare and, in almost all cases, secondary to contiguous lymph node disease [5, 12]. Because the pancreas has no definable capsule, it may be difficult to distinguish adjacent LN disease from intrinsic pancreatic infiltration [5].

The pancreas is involved in about 30% of cases of NHL, usually from contiguous nodal infiltration [2, 12]. The patterns of involvement include a circumscribed mass and diffuse glandular enlargement mimicking acute pancreatitis. Although bile duct obstruction may occur, moderate-to-severe dilatation of the main pancreatic duct is uncommon [12].

Vascular invasion, pancreatic atrophy distal to the tumor, and tumor calcification and necrosis are unusual at initial presentation. These features can help to differentiate pancreatic lymphoma from adenocarcinoma [2].

**Figure 25:**
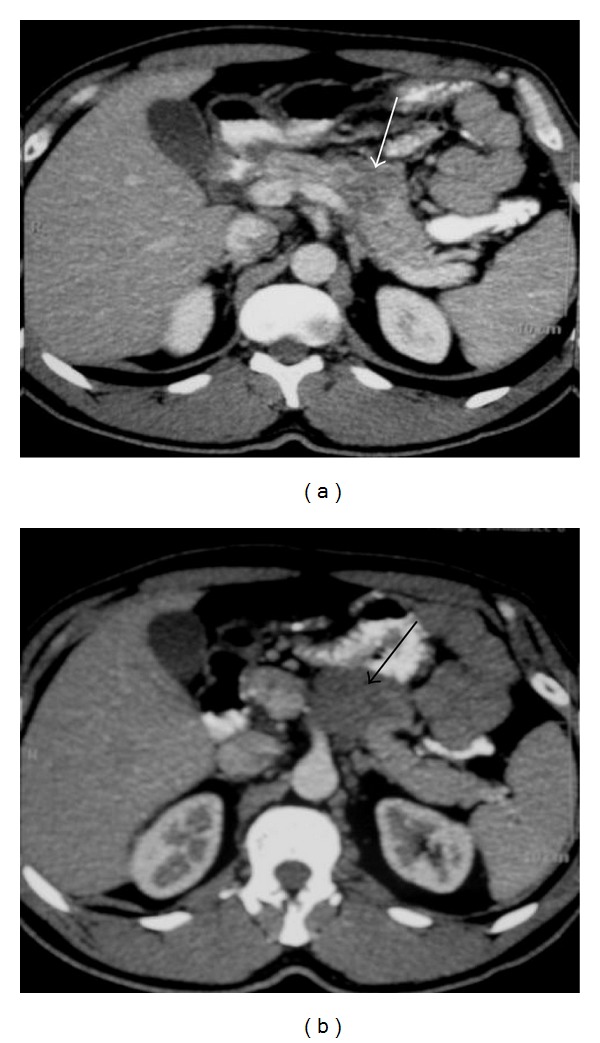
CT images of a 44-year-old man with pancreatic lymphoma. (a) Axial contrast-enhanced abdominal CT shows hypodense lesion involving the body of the pancreas (arrow). (b) Axial contrast-enhanced CT at a lower level demonstrates the lobulated hypodense mass (arrow).

**Figure 26:**
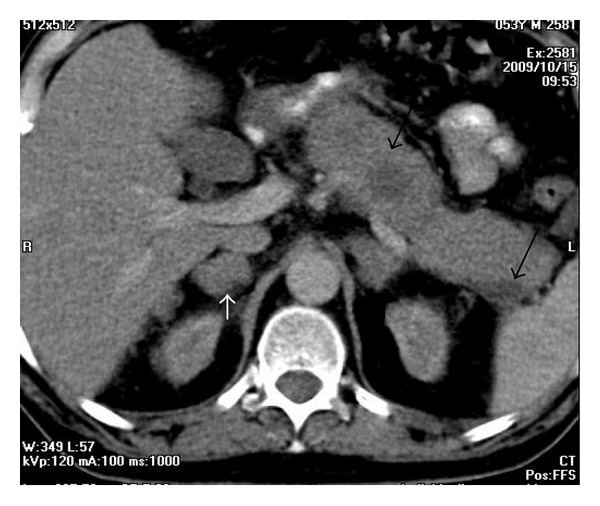
Lymphoma involving pancreas and adrenals. Axial contrast-enhanced CT demonstrates enlarged pancreas with low-attenuation nodules (arrows) and enlarged adrenal glands (small arrows).

On CT, two different morphologic patterns are seen: a localized, well-circumscribed tumoral form (Figure 25) and diffuse enlargement infiltrating (Figure 26) or replacing most of the pancreatic gland with gland enlargement and irregular infiltration of the peripancreatic fat [12].

## 8. Genitourinary Tract

Intrinsic involvement of genitourinary (GU) organ systems at presentation is rare [5]. The kidney is the most commonly involved part of the GU tract [2, 13].

### 8.1. Kidney

**Figure 27:**
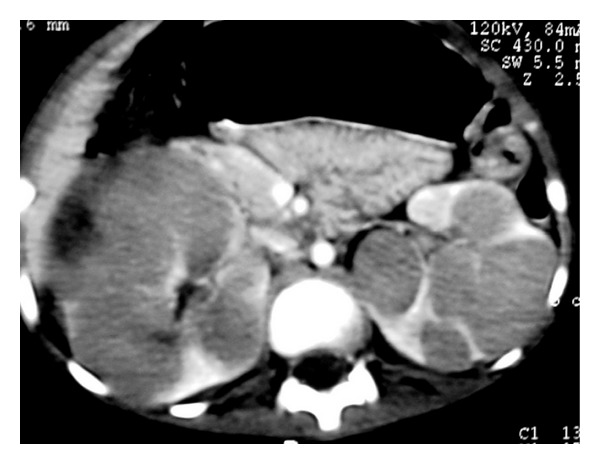
Burkitt lymphoma. Axial contrast-enhanced abdominal CT demonstrates multiple hypodense lesions involving the kidneys.

**Figure 28:**
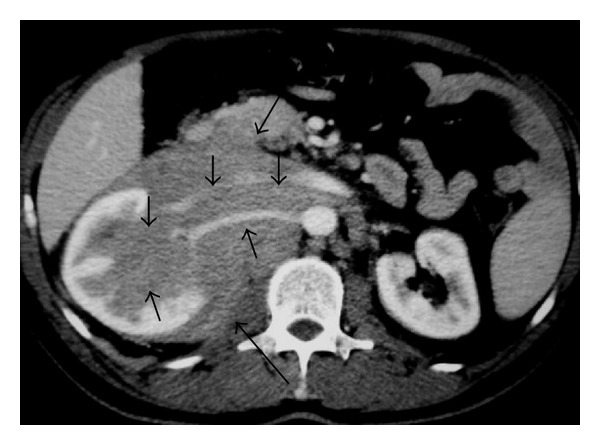
NHL. Axial contrast-enhanced CT reveals soft-tissue mass involving the right kidney (long arrows) with lymphomatous infiltration of the renal sinus and encasement of vessels (short arrows).

**Figure 29:**
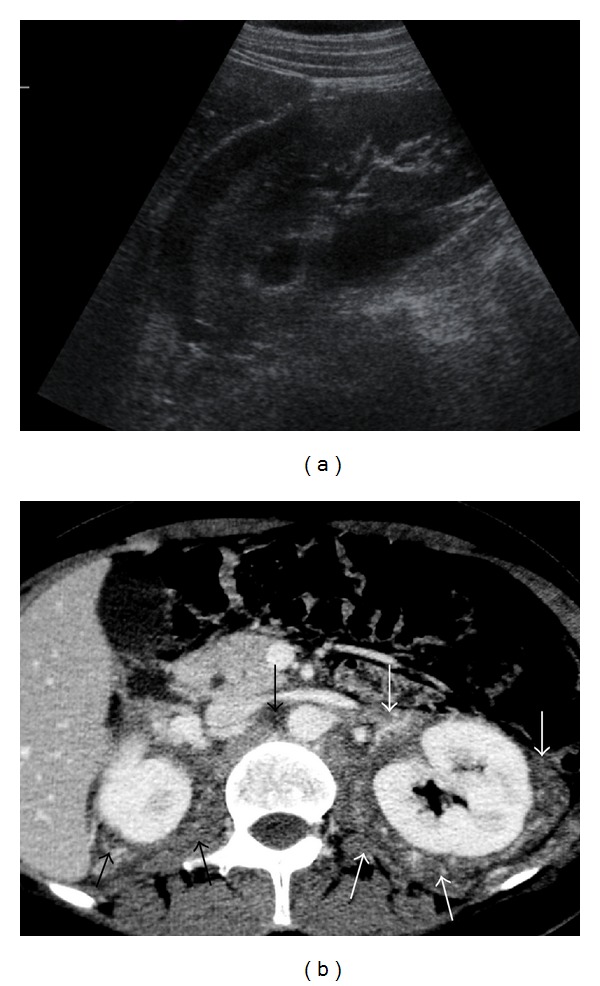
NHL. (a) Right kidney sonogram shows hypoechoic perinephric mass (arrows). (b) Axial contrast-enhanced CT scan shows a large soft-tissue mass (arrows) infiltrating the retroperitoneum and extending into the perinephric space bilaterally.

Renal involvement is extremely rare, with HD being rather perirenal and with radiologic appearance often consisting of invasion of the perirenal space by HD without renal parenchymal involvement [5]. Renal involvement occurs in 3–8% of patients with lymphoma. The patterns of renal involvement, in descending order of frequency, include multiple circumscribed masses (Figure 27), direct infiltration from adjacent nodes, a solitary mass, an isolated perinephric mass (Figure 28), and diffuse infiltration (Figure 29) [2, 13].

**Figure 30:**
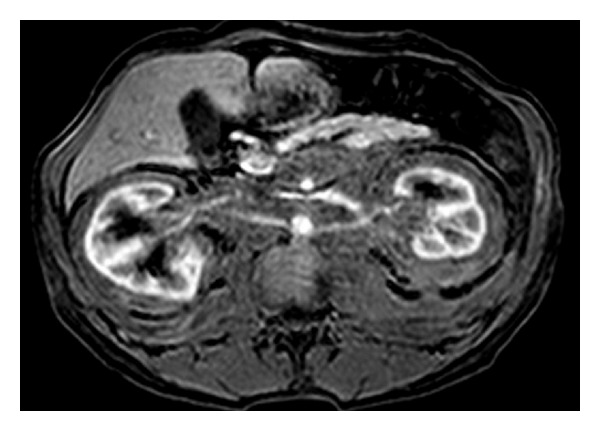
Lymphoma involving both kidneys. Axial gadolinium-enhanced T1-WI demonstrates lymphomatous infiltration of the retroperitoneum with extension to perinephric space bilaterally.

Despite peripelvic lymphoma encasing renal hilar structures, the vessels often remain patent, and there is often minimal hydronephrosis (Figure 30) which helps to differentiate peripelvic lymphoma from transitional cell carcinoma or metastases [2]. 

Renal cell carcinomas can often be differentiated from lymphoma by their hypervascular enhancement pattern [2]. 

### 8.2. Ureter

The ureter is often affected by involved retroperitoneal nodes, but primary involvement of the ureter by lymphoma is rare.

### 8.3. Bladder

Bladder involvement is also extremely rare [5]. About 8% of patients with lymphoma have bladder involvement at autopsy. The patterns of bladder involvement include circumscribed solitary or multiple masses and diffuse infiltration [2].

### 8.4. Testis

Testicular lymphoma is a rare and deadly disease representing 1% to 2% of all NH and approximately 5% of all testicular neoplasms. The most common form of testicular lymphoma is diffuse large B-cell lymphoma. Secondary involvement of the testis by NHL is more common than primary extranodal disease [14].

Lymphoma is the most common testicular tumor in older men; bilateral involvement occurs in 38% of cases. The patterns of testicular involvement include focal masses and diffuse infiltration with or without testicular enlargement [2, 14].

## 9. Adrenal Gland

**Figure 31:**
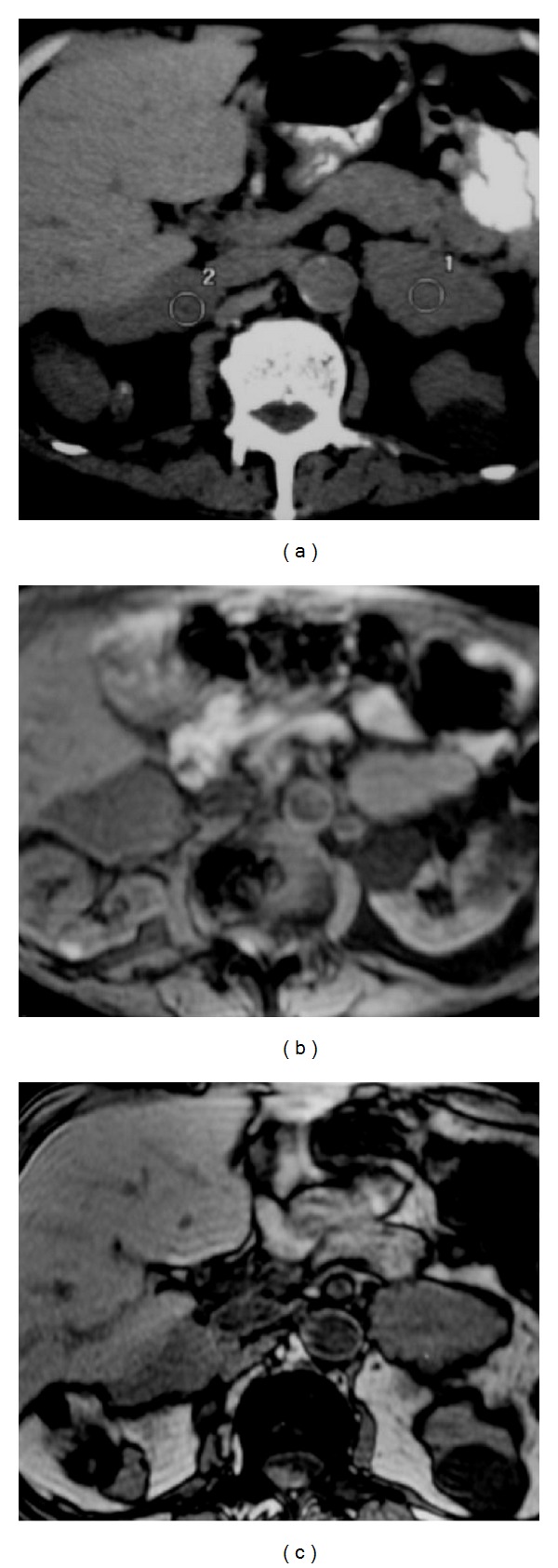
Adrenal lymphoma. Axial unenhanced CT (a) shows irregular large bilateral adrenal glands. MR axial images (b) in phase and (c) out of phase demonstrate enlarged adrenal glands.

The adrenal gland is involved in about 4% of cases of NHL. Bilateral adrenal involvement occurs in approximately 50% of these cases (Figure 31). The patterns of involvement include a rounded circumscribed homogeneous mass and an enlarged adrenal gland that maintains its normal shape [2].

## 10. Peritoneal Cavity

**Figure 32:**
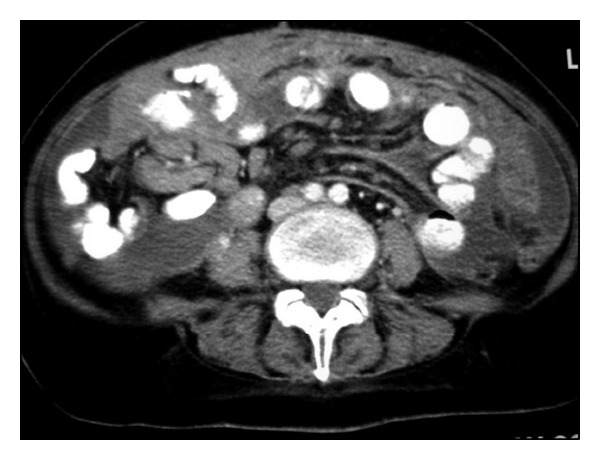
Patient with peritoneal lymphomatosis. Axial contrast-enhanced CT image shows ascites and infiltration of mesenteric fat.

Peritoneal lymphomatosis is a rare clinical presentation that is often associated with high-grade primary gastrointestinal NHL and is radiologically indistinguishable from peritoneal carcinomatosis [2, 15]. The patterns of involvement include discrete nodules, a diffuse infiltrative mass, and ascites (Figure 32). Exudative ascites from peritoneal lymphomatosis shows high attenuation because of the increased proteinaceous content. Diffuse lymphomatous infiltration of the mesentery produces a stellate appearance of the mesentery and causes fixation of the small bowel loops [2]. 

## 11. Biliary Tract

Lymphomatous involvement of the biliary tree is rare.

### 11.1. Gallbladder

The patterns of involvement of the gallbladder include an intraluminal polypoidal mass, a large mass replacing the gallbladder, and diffuse mural thickening [2].

### 11.2. Bile Ducts

The patterns of involvement of the bile ducts include a biliary stricture mimicking cholangiocarcinoma and a focal mass [2]. 

## 12. Abdominal Wall

Lymphoma may involve the abdominal wall by direct extension from bone or may occur separately in the muscle, subcutaneous fat, or skin from hematogenous spread [2]. 

## 13. AIDS-Related Lymphomas

In 1987, the Centers for Disease Control and Prevention (CDC) included several histologic types of high-grade and intermediate-grade NHL in a list of diseases that indicate a diagnosis of AIDS in patients with laboratory evidence of HIV infection [16]. Lymphoma is the second most common neoplasm associated with AIDS. AIDS-related lymphomas (ARL) have a number of highly unusual features when compared with non-ARL, including involvement of the bone marrow and skin. In addition, ARL have a striking predilection for extranodal areas of involvement, and the GI tract is the most common extranodal site [15]. ARL may affect any abdominal organ, most commonly LN, the GI tract, liver, kidney, adrenal gland, omentum, and abdominal wall.

### 13.1. Liver

Focal hepatic masses of lymphoma are much more common in AIDS patients than in the non-AIDS population. The appearance may vary from one or a few large low-attenuation masses to multiple small nodules. Hepatomegaly is common in patients with AIDS, and occasionally a large liver may harbor lymphoma without focal lesions evident on CT [16, 17].

### 13.2. Spleen

Focal splenic lesions are also seen more commonly in ARL than in lymphoma without AIDS. Single or multiple low-attenuation foci may be present. Splenomegaly is quite common in AIDS and is not predictive of involvement with lymphoma [17].

### 13.3. Lymph Nodes

Adenopathy, especially retroperitoneal and mesenteric, is a common manifestation of abdominal ARL. Suspicion of neoplasm or specific infection is limited to patients with larger nodes, nodes in other locations, or large clusters of smaller nodes. If nodes have low-attenuation centers, mycobacterial infection, rather than lymphoma, is likely [17].

### 13.4. Omentum

The omentum may be grossly infiltrated with lymphoma as one manifestation of peritoneal lymphomatosis [16, 17].

### 13.5. GI Tract

Focal masses of ARL may be seen in the stomach, small bowel, or colon. Masses in the GI tract may be isolated findings, but evidence of disease elsewhere is common.

The radiographic, CT, and barium examination features of intrinsic bowel involvement are similar in patients with AIDS and immunocompetent patients, and no gross morphologic differences among the different histologic types are found [17, 18].

### 13.6. Kidney

Focal masses of ARL in the kidney are not as common as in the liver or spleen. One or more renal masses may be seen. Masses may be sharply circumscribed or may infiltrate into the renal sinus or surrounding tissues [16, 17].

### 13.7. Retroperitoneum

A grossly enlarged psoas muscle may represent a primary manifestation of ARL [16].

Although familiarity with the imaging features of lymphoma is important, a definitive diagnosis requires a biopsy. In patients with known disease, the goals of imaging are staging, evaluation of response to therapy, and identification of new or recurrent disease or of complications of therapy. In patients without known disease, imaging permits a provisional diagnosis.
